# Two-item PROMIS® global physical and mental health scales

**DOI:** 10.1186/s41687-017-0003-8

**Published:** 2017-09-12

**Authors:** Ron D. Hays, Benjamin D. Schalet, Karen L. Spritzer, David Cella

**Affiliations:** 1 Division of General Internal Medicine & Health Services Research, Center for Maximizing Outcomes and Research on Effectiveness (C-MORE), University of California, 911 Broxton Avenue, Los Angeles, CA 90024 USA; 2 Northwestern University, Feinberg School of Medicine, 633 N. St Clair, 19th Floor, Chicago, IL 60611 USA

**Keywords:** PROMIS®, Global health, Patient-reported outcomes

## Abstract

**Background:**

Self-reports of health provide useful information about function and well-being that can improve communication between patients and clinicians. Global health items provide summary information that are predictive of health care utilization and mortality. There is a need for parsimonious global health scales for use in large sample surveys. This study evaluates the reliability and validity of brief measures of global physical health and mental health in the Patient Reported Outcomes Measurement and Information System (PROMIS®) project.

**Methods:**

A total of 21,133 persons included in the PROMIS development sample: 52% female; 82% White, 9% Black, 9% Hispanic; median age of 50 years. We identified two global physical health items (GPH-2) and two global mental health items (GMH-2) with highest discrimination parameters and compared their reliabilities and construct validity to that of the original 4-item scales (GPH-4 and GMH-4) and a single global health item (Global01).

**Results:**

Internal consistency reliability was 0.73 for the GPH-2 (versus 0.81 for the GPH-4) and 0.81 for the GMH-2 (versus 0.86 for the GMH-4). Marginal reliabilities were 0.55 for *Global01*, 0.70 for GPH-2, 0.79 for GPH-4, 0.80 for GMH-2, and 0.86 for GMH-4. The product-moment correlation between the GPH-2 and GPH-4 was 0.94 and between GMH-2 and GMH-4 was 0.97. The 2-item and 4-item versions of the scales had similar correlations with PROMIS domain scores, the EQ-5D-3L and comorbidities, but the 4-item scales were more strongly correlated with these measures.

**Conclusions:**

Adding a single item to a large cross-sectional population survey can cost as much as $100,000. The 2-item variants of the PROMIS global health scales reduce the cost of use on national surveys by 50%, a substantial cost savings. These briefer scales are also more practical for use in clinical practice. The 2-item versions of the PROMIS global health scales display adequate reliability for group comparisons and their associations with other indicators of health are similar to that of the original 4-item scales. The briefer scales are psychometrically sound and reduce burden of survey administration.

## Background

The Patient-Reported Outcomes Measurement Information System (PROMIS®) is a National Institutes of Health initiative to develop state-of-the-science measures that assess function and well-being in the physical, mental and social domains of health. PROMIS goals include using these measures as indicators of health care outcomes that may guide reduction of health care disparities and improvement of population health in the U.S. [1]. These measures are useful in screening for disability and in improving communication between patients and clinicians [2]. In addition, self-reported health is predictive of health care utilization and subsequent mortality [3].

Global health items assess overall health. PROMIS global health items include global ratings of five primary domains (physical function, fatigue, pain, emotional distress, and social health) as well as perceptions of general health that cut across domains [4]. Global items allow respondents to weigh together different aspects of health to arrive at a “bottom-line” indicator of their health. Four-item global physical health (GPH-4) and global mental health (GMH-4) scales were developed in PROMIS® that had internal consistency reliability coefficients of 0.79 and 0.86, respectively.

Despite the parsimony of the GPH-4 and GMH-4, there are applications where even fewer global health items are desired. For example, adding a single item to a large population survey can cost as much as $100,000. Indeed, the most widely used global health measure is a single item: In general, how would you rate your health: excellent, very good, good, fair, or poor? [5] This item (*Global01*) correlated strongly with the GPH-4 but its reliability was considerably lower than that of the GPH-4 [6]. When considering briefer versions of multi-item scales, comparability of scores produced from the shorter measure needs to be demonstrated and tradeoffs carefully considered [7].

This study identifies 2-item variants of the PROMIS global physical and mental health scales (GPH-2 and GMH-2) and compares their psychometric properties to the GPH-4, GMH-4, and the *Global01* item*.*

## Methods

### Sample

The data were collected in 2007 and 2008 and consisted of 21,133 individuals, of whom 19,601 were members of the YouGovPolimetrix panel sample, while 1532 were recruited at medical sites (University of North Carolina, Stanford, Pittsburgh, and Duke) [1]. The sample was 52% female, had median age of 50 years, 82% White (non-Hispanic), 9% Black (non-Hispanic), and 9% Hispanic. Three percent of this sample had less than a high school education, 16% were high school graduates, and 43% had educational attainment beyond high school. While those with lower levels of educational attainment were underrepresented [8], each global health item response option was selected by at least 100 respondents. In addition, equivalence testing showed similarly between the PROMIS general population and national norms related to body mass index and self-rated health [9].

### PROMIS measures

The PROMIS Global Health (v 1.2) instrument consists of ten global health items that represent five core PROMIS domains (physical function, pain, fatigue, emotional distress, social health). Four items are used to assess global physical health. Three of these are administered using five-category response scales, and one item (rating of pain on average) uses a response scale of 0–10 that is recoded to five categories (0 = 1; 1-3 = 2; 4-6 = 3; 7-9 = 4; 10 = 5):

1. In general, how would you rate your physical health? Excellent, Very Good, Good, Fair, Poor
2. To what extent are you able to carry out your everyday physical activities such as walking, climbing stairs, carrying groceries, or moving a chair? Completely, Mostly, Moderately, A little, Not at all
3. In the past 7 days, how would you rate your pain on average? Scale of 0 to 10, where 0 = no pain and 10 = worst pain imaginable
4. In the past 7 days, how would you rate your fatigue on average? None, Mild, Moderate, Severe, Very severe

Four items are used to assess global mental health, all of which are administered using five-category response scales:

1. In general, would you say your quality of life is: Excellent, Very Good, Good, Fair, Poor
2. In general, how would you rate your mental health, including your mood and your ability to think? Excellent, Very Good, Good, Fair, Poor
3. In general, how would you rate your satisfaction with social activities and relationships? Excellent, Very Good, Good, Fair, Poor
4. How often have you been bothered by emotional problems? Never, Rarely, Sometimes, Often, Always

The dataset also included PROMIS version 1.0 measures of physical function, pain behavior, pain interference, fatigue, anxiety, anger, depressive symptoms, satisfaction with participation in discretionary social activities, satisfaction with participation in social roles, as well as self-reported chronic conditions and the EQ-5D-3L [10].

The Evanston Northwestern Healthcare institutional review board reviewed and approved the study.

### Analysis plan

We selected 2 of 4 items from both the GPH-4 and GMH-4 scales for psychometric evaluation (GPH-2 and GMH-2) that had the highest discrimination parameters [11], indicating they best represented the underlying construct. The GPH-2 items are: 1) *Global03*: In general, how would you rate your physical health? 2) *Global06*: To what extent are you able to carry out your everyday physical activities such as walking, climbing stairs, carrying groceries, or moving a chair? The GMH-2 items are: 1) *Global04*: In general, how would you rate your mental health, including your mood and your ability to think? 2) *Global05*: In general, how would you rate your satisfaction with your social activities and relationships?

We provide mean scores, internal consistency reliability [12], and marginal reliability of the GPH-4, GPH-2, *Global01*, GMH-4 and GMH-2 scales. Marginal (empirical) reliability was estimated by calculating the ratio of the average of the squared standard errors of observed expected a-posteriori (EAP) scores over the observed EAP score variance, and subtracting that ratio from one. In addition, we estimated product-moment correlations of the 2-item scales (GPH-2 and GMH-2) and the single item (*Global01*) with the original 4-item (GPH-4 and GMH-4) scales. We also evaluated construct validity using product-moment correlations with other measures included in the study: PROMIS physical function, pain behavior, pain interference, fatigue, anxiety, anger, depressive symptoms, social discretionary and social roles domains, EQ-5D-3L, and count of number of 25 self-reported chronic conditions: high blood pressure (hypertension), chest pain (angina), hardening of the arteries (coronary artery disease), heart failure or congestive heart failure, heart attack (myocardial infarction), stroke or transient ischemic attack (TIA), liver disease, hepatitis or cirrhosis, kidney disease, arthritis or rheumatism, osteoarthritis or degenerative arthritis, migraines or severe headaches, asthma, chronic lung disease (COPD), chronic bronchitis or emphysema, diabetes or high blood sugar or sugar in your urine, cancer (other than non-melanoma skin cancer), depression, anxiety, alcohol or drug problem, sleep disorder, HIV or AIDS, spinal cord injury, multiple sclerosis, Parkinson’s Disease, epilepsy, and ALS (amyotrophic lateral sclerosis). We also included a count of the number of those conditions that were reported to limit the respondent’s current activities. Both number of conditions variables were recoded to 0, 1, 2, 3, 4, or 5 or more conditions.

## Results

As seen in Table 1, means ranged from 49.10 to 49.41 for the GPH-4, GPH-2 and *Global01*, while means were 49.85 and 49.91 for the GMH-4 and GMH-2, respectively. Coefficient alpha for the GPH-2 was 0.73 (versus 0.81 for the GPH-4) and 0.81 for the GMH-2 (versus 0.86 for GMH-4). Marginal reliabilities were 0.79 for GPH-4, 0.70 for GPH-2, 0.55 for *Global01*, 0.86 for GMH-4, and 0.80 for GMH-2. The product-moment correlation of the GPH-2 with the GPH-4 was 0.94 and between the GMH-2 and GMH-4 was 0.97. The single item (*Global01*) correlated 0.80 with GPH-4 and 0.60 with GMH-4.

**Table 1:** Means, Standard Deviations, and Reliability Estimates for PROMIS Global Health Scales

	Mean	SD	Lower Quartile	Upper Quartile	Quartile Range	Alpha	Marginal Reliability
GPH-4	49.10	9.21	42.84	54.54	11.70	0.81	0.79
GPH-2	49.21	8.71	44.32	56.03	11.71	0.73	0.70
*Global01*	49.41	7.56	46.77	54.26	7.49	NA	0.55
GMH-4	49.85	9.56	43.32	56.68	13.36	0.86	0.86
GMH-2	49.91	9.18	44.12	56.41	12.29	0.81	0.80

*GPH-4* 4-item global physical health scale, *GPH-2* 2-item global physical health scale, *Global01* Single general health rating item, *GMH-4* 4-item global mental health scale, *GMH-2* 2-item global mental health scale, *SD* Standard deviation, *Alpha* Coefficient alpha, *NA* Not applicable, Marginal reliability is one minus the ratio of the average of the squared standard errors of observed expected a-posteriori (EAP) scores over the observed EAP score variance

Correlations of the global health scales with other PROMIS measures, the EQ-5D-3L, and the count of chronic condition variables are given in Table 2. The 2-item variants of the global health scales had the same pattern of correlations with other measures but they tended to be slightly smaller in magnitude. The largest correlation of the *Global01*, GPH-2 and GHP-4 was with physical function, and the largest correlation for the GMH-4 and GMH-2 was with depressive symptoms.

**Table 2:** Product-moment correlations with PROMIS Global Health Scales

	GPH-4	GPH-2	*Global01*	GMH-4	GMH-2
Physical function	0.78	0.76	0.62	0.43	0.38
Pain behavior	−0.64	−0.53	−0.47	−0.41	−0.37
Pain interference	−0.73	−0.64	−0.55	−0.50	−0.45
Fatigue	−0.72	−0.60	−0.56	−0.66	−0.60
Anxiety	−0.46	−0.38	−0.37	−0.64	−0.60
Anger	−0.32	−0.26	−0.27	−0.49	−0.46
Depressive symptoms	−0.46	−0.39	−0.39	−0.69	−0.65
Social discretionary	0.52	0.47	0.42	0.60	0.57
Social roles	0.62	0.57	0.50	0.60	0.56
EQ-5D-3L	0.74	0.66	0.57	0.56	0.51
Chronic conditions^a^	−0.53	−0.51	−0.48	−0.33	−0.30
Conditions that are reported to limit current activities^a^	−0.60	−0.58	−0.51	−0.46	−0.42

*GPH-4* 4-item global physical health scale, *GPH-2* 2-item global physical health scale, *Global01* Single general health rating item, *GMH-4* 4-item global mental health scale, *GMH-2* 2-item global mental health scale. All correlations statistically significant (*p* < .001)

^a^Count of 25 conditions scored as 0, 1, 2, 3, 4, and 5 or more. The conditions were: high blood pressure (hypertension), chest pain (angina), hardening of the arteries (coronary artery disease), heart failure or congestive heart failure, heart attack (myocardial infarction), stroke or transient ischemic attack (TIA), liver disease, hepatitis or cirrhosis, kidney disease, arthritis or rheumatism, osteoarthritis or degenerative arthritis, migraines or severe headaches, asthma, chronic lung disease (COPD), chronic bronchitis or emphysema, diabetes or high blood sugar or sugar in your urine, cancer (other than non-melanoma skin cancer), depression, anxiety, alcohol or drug problem, sleep disorder, HIV or AIDS, spinal cord injury, multiple sclerosis, Parkinson’s Disease, epilepsy, and ALS (amyotrophic lateral sclerosis)

## Discussion and conclusions

The PROMIS Global Health (v 1.2) instrument contains one physical and one mental health scale. These scales were developed to be parsimonious generic self-report measures of health, consisting of 4 items each [11]. This study evaluates even briefer (2-item) versions of the scales. The 2-item scales had lower but acceptable levels of reliability (0.70 or above) for group-level comparisons [13]. These new variants also had similar but slightly smaller correlations with PROMIS health domain scores, the EQ-5D-3L, and chronic conditions. In addition, the benefit of using just two items rather than one is evident by comparing the GPH-2 with the “In general, how would you rate your health?” item (G*lobal01*) evaluated previously [6]. Marginal reliability was larger for the GPH-2 than for *Global01*. In addition, GPH-2 correlated more strongly with GPH-4, the PROMIS domain scores, the EQ-5D-3L, and count of chronic conditions than *Global01* did. The global physical health forms are available for download, scoring, and electronic administration at http://www.healthmeasures.net/search-view-measures. The full names of the current versions are PROMIS Scale v1.2-Global Health Physical 2a and PROMIS Scale v1.2-Global Health Mental 2a.

A major advantage of these new 2-item scales is that they reduce the cost of use on national surveys by 50%. This represents substantial cost savings because adding a single item to a large cross-sectional population survey can cost as much as $100,000. Hence, briefer scales reduce the cost and burden of measuring global health. Thus, the two-item versions of global physical and mental health appear to be good options for estimating self-reported health in large sample surveys, including population-based public health surveys. These briefer variants of the PROMIS global health scales may also be useful for screening of patients in clinical practices analogous to what is done with the Dartmouth COOP charts [14]. Simple tools such as these brief measures increase the likelihood of successful integration and institutionalization by practices [15].
